# Sequelae in COVID‐19 patients 3 months after hospital discharge or completion of self‐isolation

**DOI:** 10.1002/hsr2.444

**Published:** 2021-12-21

**Authors:** Desdiani Desdiani, Auditya Purwandini Sutarto, Alfathul Nur Kharisma, Hera Safitri, Amalia Fitri Hakim, Salsabila Hanifa Rusyda

**Affiliations:** ^1^ Faculty of Medicine Universitas Sultan Ageng Tirtayasa Cilegon Indonesia; ^2^ Department of Pulmonology and Respiratory Medicine Bhayangkara Brimob Hospital Depok Indonesia; ^3^ Department of Industrial Engineering Universitas Qomaruddin Gresik Gresik Indonesia; ^4^ Faculty of Medicine Universitas Sriwijaya Palembang Indonesia; ^5^ Faculty of Medicine Universitas Andalas Padang Indonesia

## 1 INTRODUCTION

Many patients experience post‐COVID‐19 sequelae that lead to impaired quality of life after recovering from COVID‐19.^1^ To date, there have been 1 657 035 cases of COVID‐19, with 45 116 dead and 1 511 417 recovered in Indonesia.^2^ We assessed sequelae in COVID‐19 patients and most common sequelae, both those who had self‐isolated and those who were hospitalized, who had recovered for more than 3 months after negative polymerase chain reaction (PCR) swab results. COVID‐19 sequelae are persistent symptomatology, and outcomes after hospital discharge or completion of self‐isolation with a wide and multifaceted range of clinical manifestations were identified, including respiratory, gastrointestinal, neurological, cardiovascular symptoms, and other organ manifestations.^1^

## 2 METHODS

This study by the Faculty of Medicine, Sultan Ageng Tirtayasa University assessed the sequelae of adult patients who confirmed positive for COVID‐19 infection based on PCR examination, who had undergone self‐isolation or hospitalization and had been declared recovered for more than 3 months. These sequelae are persistent symptoms that develop during or following a confirmed case of COVID‐19 and that continue for >28 days. The patients were retrospectively included in this study. Informed consent was signed by the patients, and this study was approved by the ethics committee of Bhayangkara Brimob Hospital. This study follows the Strengthening the Reporting of Observational Studies in Epidemiology (STROBE) reporting guideline. The sequelae of COVID‐19 were those reported since the patient was declared negative by a PCR swab and had completed self‐isolation or hospitalization for 10 days plus 3 days at the hospital, as recommended by the World Health Organization and the local Ministry of Health. The data collected were self‐reported symptoms that appeared from when the patient was declared recovered from COVID‐19 until 90 days later. The data were obtained from various cities in Indonesia.

A total of 214 patients who had been declared recovered from COVID‐19 were contacted between January and March 2021 to complete a single electronic questionnaire between 1 and 3 months after being declared recovered. Thirteen participants who did not complete the questionnaire were not included in the analysis. We performed only descriptive analysis due to the small number of participants. Data analysis was conducted using SPSS 23.

## 3 RESULTS

A total of 201 participants took part from Indonesia and were of Asian ethnicity with an average age of 39.35 years old; the participants included 109 (54.2%) males and 92 (45.8%) females with confirmed COVID‐19 infection. A total of 19 COVID‐19 patients (9.45%) had had no symptoms, 58 (28.85%) patients had undergone self‐isolation, and 143 (71%) had required hospitalization (Table 1). Hypertension and diabetes mellitus were the most common comorbidities found in 31 patients (15.42%). A total of 17 (8.46%) participants were active smokers.

**TABLE 1:** Demographic and clinical characteristics of the study participants

Characteristics	No. %			
	Total recovered individuals (n = 201)	Inpatient (n = 143)	Outpatient	
			Symptomatic (n = 39)	Asymptomatic individuals (n = 19)
Age, mean (SD)	39.35 (11.56)	39.1	40.2	39.6
Sex				
Women	92 (45.8)	57 (39.9)	27 (69.2)	8 (42.1)
Men	109 (54.2)	86 (60.1)	12 (30.8)	11 (57.9)
BMI, mean (SD)	26.3	26.3	26.5	26.3
Comorbidities				
Hypertension	19 (9.45)	18 (12.59)	1 (2.6)	0 (0.0)
Diabetes	12 (5.97)	9 (6.29)	3 (7.7)	0 (0.0)
Active smoker	17 (8.46)	13 (9.09)	1 (2.6)	3 (15.8)
Duration of persistent symptoms (mo)				
0	80 (39.8)	56 (39.2)	17 (43.6)	7 (36.8)
1 to 2	38 (18.9)	28 (19.6)	10 (25.6)	0 (0.0)
≥3	9 (4.5)	5 (3.5)	2 (5.12)	2 (10.5)
Missing	74 (36.8)	54 (37.8)	10 (25.6)	10 (52.6)
Worse quality of life	47 (23.4)	34 (23.8)	12 (30.8)	1 (5.3)
Symptoms (post‐COVID follow‐up)	127 (63.18)	89 (62.2)	27 (69.2)	11 (57.9)
Excessive fatigue/tiredness	69 (54.3)	44 (49.4)	21 (53.8)	4 (21.0)
Dyspnea/shortness of breath	26 (20.47)	18 (20.2)	5 (12.8)	3 (15.8)
Sleep disorders	25 (16.68)	17 (19.1)	7 (17.9)	1 (5.3)
Emotional instability	22 (17.3)	18 (20.2)	4 (10.3)	0 (0.0)
Difficulty concentrating	34 (26.77)	16 (17.97)	14 (35.9)	4 (21.0)
Digestive problems	25 (19.68)	16 (17.97)	8 (20.5)	1 (5.3)
Loss of appetite	9 (7.09)	7 (7.86)	2 (5.1)	0 (0.0)
Loss of smell	10 (7.84)	5 (5.61)	3 (7.7)	2 (10.5)
Muscle pain	24 (11.94)	23 (25.84)	1 (2.6)	0 (0.0)
Sore throat	8 (6.3)	7 (7.86)	1 (2.6)	0 (0.0)
Cough	33 (25.98)	25 (28.1)	7 (17.9)	1 (5.3)
Cold	19 (14.96)	16 (17.97)	3 (7.7)	0 (0.0)
Diarrhea	2 (1.57)	1 (1.1)	0 (0.0)	1 (5.3)
Earache	1 (0.79)	0 (0.0)	1 (2.6)	0 (0.0)
Sweating	15 (11.81)	14 (15.7)	1 (2.6)	0 (0.0)
Burning sensation on the skin	8 (6.3)	5 (5.6)	3 (7.7)	0 (0.0)
Chills	2 (1.57)	2 (2.25)	0 (0.0)	0 (0.0)
Felt feverish	13 (10.23)	13 (14.6)	0 (0.0)	0 (0.0)
Nausea	7 (5.51)	7 (7.86)	0 (0.0)	0 (0.0)

Abbreviation: BMI, body mass index.

Overall, 127 of the 201 (63.18%) patients reported having experienced sequelae (Table 1). Of these 127 patients, sequelae were reported by 61 patients (48.03%) aged 18 to 39 years, 63 patients (49.60%) aged 40 to 64 years, and three patients (2.36%) aged ≥65 years. A total of 89 participants (62.2%) with sequelae had been hospitalized, and 38 (65.51%) had independently self‐isolated. Sequelae were reported by 19 (9.45%) patients who initially had no symptoms, but after PCR was negative and they had completed self‐isolation, persistent symptoms appeared and were believed not to derive from another illness condition. Some symptoms appeared to be dominant in the patient's report. A total of 15 of 31 (48.39%) patients with hypertension or diabetes (comorbidities) had persistent symptoms to date.

**FIGURE 1:**
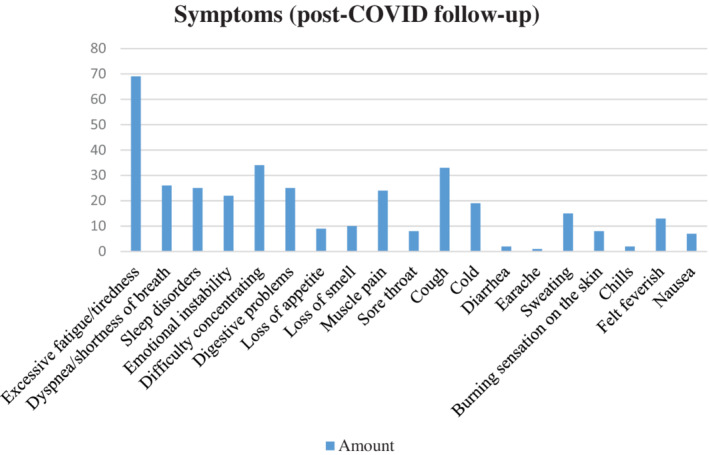
Symptoms (post‐COVID follow‐up)

**FIGURE 2:**
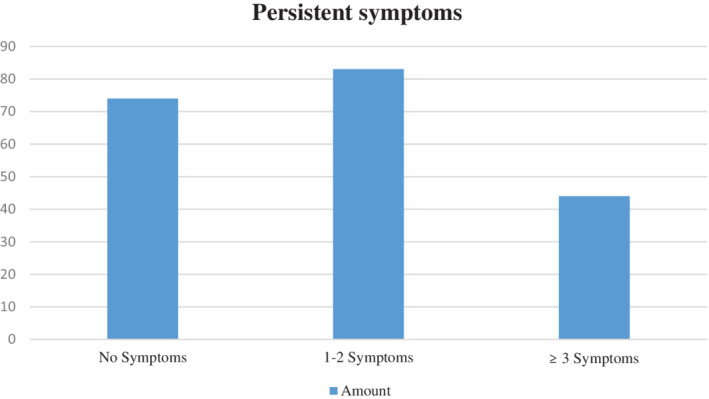
Persistent symptoms

The most frequently reported sequelae were excessive fatigue (69/127 patients [54.33%]), difficulty concentrating (34/127 [26.77%]), and cough (33/127 patients [25.98%]) (Figure 1). A total of 83 (41.35%) participants experienced sequelae for 1 to 2 months, and 44 (21.9%) people experienced sequelae for 3 or more months (Table 2 and Figure 2). Overall, 47 of 201 (23.38%) participants who had undergone self‐isolation or who had been hospitalized reported a decrease in quality of life compared with three participants who had no symptoms (7.1%).

**TABLE 2:** Number of symptoms

Persistent symptoms	No. (%)
No symptoms	74 (36.8)
1‐2 symptoms	83 (41.3)
≥3 symptoms	44 (21.9)

## 4 DISCUSSION

In this study, patients were studied at 3 months after being discharged from hospital or declared recovered. A total of 127 (63.18%) patients reported sequelae that were still felt after being declared recovered with negative PCR confirmation. Eighty people (39.8%) reported sequelae lasting less than 1 month. A total of 38 people (18.9%) reported sequelae lasting around 1 to 2 months, and nine (4.5%) experienced sequelae ≥3 months after being discharged from hospital or being declared recovered. Approximately 143 (71.14%) of the participants in our study had been hospitalized for COVID‐19. Sequelae experienced for more than 1 month were reported by 33 (23.1%) of patients who were hospitalized. A study by Sudre et al stated that as many as 20.1% of participants with COVID‐19 still experienced symptoms and who had recovered from COVID‐19 for more than 28 days to more than 12 weeks.^3^

The most reported sequelae in our study were excessive fatigue (69 of 127 patients [54.33%]), difficulty concentrating (34 of 127 patients [26.77%]), and cough (33 of 127 patients [25.98%]). A total of 83 (41.3%) participants experienced 1 to 2 sequelae symptoms, and 44 (21.9%) people experienced ≥3 sequelae. Fourteen of 27 (51.85%) comorbid patients who had been hospitalized had persistent symptoms for more than 1 month. Garrigues et al revealed that the most reported persistent symptoms were fatigue (55%), shortness of breath (42%), loss of memory (34%), and impaired concentration and sleep (28% and 30.8%, respectively). Comparison of the persistent symptoms that occurred between the usual isolation‐treated patients and the patients who received ICU care was statistically significant. Many symptoms persist several months after hospitalization for COVID‐19. While there were few differences between health‐related quality of life (HRQoL) between ward and ICU patients.^4^ A study by Carfi et al revealed that 87.4% of patients reported persistent symptoms of at least one symptom, especially fatigue and shortness of breath.^5^

A decrease in quality of life was reported in this study, with 47 patients (23.4%) from the total group of participants and 34 (23.8%) of the hospitalized patients experiencing a decrease in quality of life. Logue et al also reported impaired quality of life in 30.7% of outpatients who had self‐isolated.^6^

In the case of health workers who work in the operating room, the risk of contracting COVID‐19 can have serious consequences. The importance of proper PPE use, reorganization of the operating schedule, use of safe operating techniques, and user‐friendly surgical equipment are all good ways to avoid smoke production.^7^ Surgical team members responded to the COVID‐19 pandemic with leadership and crisis management principles. Current recommendations are widely adopted in terms of organizational aspects and surgical management.^8^ The surgery departments implemented changes, including reorganizing surgical schedules, staff preparation, and the departments' outbreak response policies and suggestions for surgical techniques and risk management.^9^ Surgeons reported that their apparent lack of safety and comfort, as well as increased fatigue, may have hampered their ability to perform at their best throughout surgery.^10^

This study is limited because of its small sample size. Although the respondents involved in this study came from several regions in Indonesia, the locations were not included in the analysis. There is potential for bias in reporting symptoms during the post‐recovery period of up to 3 months. This study only analyzed symptoms that were observed and followed up with questionnaire completed by the patients, more than 3 months after recovery and which were not observed during hospitalization or self‐isolation. This study also did not differentiate between isolation room in hospital and ICU inpatient care but only between patients who self‐isolated and hospitalized patients. Our study shows that the sequelae of COVID‐19 are still felt by patients even after being discharged from hospital when the PCR results are negative, thus affecting the participants' quality of life. This study is an initial report of the sequelae that are still felt by patients after recovery from COVID‐19 in Indonesia.

## CONFLICT OF INTEREST

The authors declare no conflict of interest for this article.

## AUTHOR CONTRIBUTIONS

Conceptualization: Desdiani Desdiani

Formal analysis: Desdiani Desdiani, Auditya Purwandini Sutarto

Investigation: Alfathul Nur Kharisma, Hera Safitri, Amalia Fitri Hakim, Salsabila Hanifa Rusyda

Project Administration: Desdiani Desdiani

Resources: Desdiani Desdiani, Auditya Purwandini Sutarto, Hera Safitri

Supervision: Desdiani Desdiani

Visualization: Hera Safitri, Amalia Fitri Hakim, Salsabila Hanifa Rusyda

Writing—Original Draft Preparation: Desdiani Desdiani, Amalia Fitri Hakim, Salsabila Hanifa Rusyda

Writing—Review and Editing: Desdiani Desdiani, Auditya Purwandini Sutarto, Amalia Fitri Hakim, Salsabila Hanifa Rusyda

All authors had read and approved the final version of the manuscript.

Desdiani Desdiani had full access to all of the data in this study and takes complete responsibility for the integrity of the data and the accuracy of the data analysis.

## TRANSPARENCY STATEMENT

Desdiani Desdiani confirms that the manuscript is an honest, accurate, and transparent account of the study being reported; that no important aspects of the study have been omitted; and that any discrepancies from the study as planned (and, if relevant, registered) have been explained.

## Data Availability

The authors confirm that the data supporting the findings of this study are available within the article and its supplementary materials.
